# A Case of Eating Disorder Diagnosed As Orthorexia Nervosa

**DOI:** 10.7759/cureus.33801

**Published:** 2023-01-15

**Authors:** Ai Yoshimura, Yoshiki Kusama, Yuka Omura, Mariko Shibata, Toshiro Maihara

**Affiliations:** 1 Department of Pediatrics, Hyogo Prefectural Amagasaki General Medical Center, Hyogo, JPN; 2 Department of Diabetes, Endocrinology, and Metabolic Diseases, Jikei University School of Medicine, Tokyo, JPN; 3 Department of Psychiatry, Hyogo Prefectural Amagasaki General Medical Center, Hyogo, JPN

**Keywords:** covid-19, dsm-ⅴ, eating disorder, anorexia nervosa (an), orthorexia nervosa

## Abstract

A 13-year-old girl presented to our hospital with chief complaints of rapid weight loss, fatigue, discomfort, chills in the extremities, and alopecia. We initially suspected anorexia nervosa (AN). However, she did not express fear of gaining weight or have a distorted perception of her weight or body shape; thus, her presentation was not typical of AN. We also suspected avoidant/restrictive food intake disorder (ARFID), but she did not exhibit any food-avoidance behaviors. However, she was obsessed with nutrition control, so we diagnosed her with orthorexia nervosa (ON). She was hospitalized, given education on proper nutrition, and her eating behavior subsequently improved. After discharge, we administered the ORTO-15, which assesses the propensity for ON, and her score met the diagnostic criteria for ON. The incidence of ON has increased during the COVID-19 pandemic. In this case, her obsession was brought about by information she read in magazines and on social media that promoted an unbalanced diet centered almost exclusively on vegetables. Pediatricians should raise awareness of misinformation regarding children’s health to ensure healthy growth.

## Introduction

Mortality rates from eating disorders are 5.2-6.5 times higher in people with anorexia nervosa (AN), 1.4-1.5 times higher in those with bulimia nervosa (BN), and 1.5-2.3 times higher in those with binge eating disorder compared with the same-aged population [1]. Thus, physicians should recognize that eating disorders can be fatal. The morbidity of eating disorders is higher in the population aged ≥10 years [2]; therefore, eating disorders do not always occur in adults. Thus, pediatricians should know the ways to diagnose and treat eating disorders. In 2013, the 5th edition of the Diagnostic and Statistical Manual of Mental Disorders (DSM-5) was published, and the classification of eating disorders was updated. However, the American Psychiatric Society has not yet recognized orthorexia nervosa (ON) and is therefore not included in the DSM-5. Herein, we report a patient with an eating disorder who met the diagnostic criteria for ON.

## Case presentation

A previously healthy 13-year-old Japanese girl in junior high school with no diagnosed developmental disability presented to our hospital with severe weight loss. She was quiet and had made no apparent trouble between friends and teachers during elementary school. Her school grades in junior high school were generally fair. She has no siblings, and her parents did not have any histories of eating disorders and were not interested in healthism. Five months before the hospital visit, she gradually began to lose weight. Two months before, her weight rapidly declined, she stopped menstruating, and hair loss was noted. There were no episodes of overeating, no self-induced vomiting, no use of laxatives or diuretics, and no increase in physical activities. Because she recognized lethargy, mood discomfort, and cold extremities, she visited a primary care doctor, who referred her to our hospital because of her marked emaciation. At the time of admission, her height and weight were 146.5 cm and 27.9 kg, respectively, and her BMI was 13.0. Her vital signs included a body temperature of 35.9°C, pulse rate of 83 beats per min, respiratory rate of 20 breaths per min, blood pressure of 95/61 mmHg, and oxygen saturation of 100% (on room air). Her extremities were cold, but no edema was observed. Blood tests revealed hypercholesterolemia and low free T3 (Table 1).

**Table 1:** Results of blood test on admission. AST: Aspartate aminotransferase; ALT: Alanine aminotransferase; HDLC: High-density lipoprotein cholesterol; TSH: Thyroid-stimulating hormone; FT3: Free triiodothyronine; FT4: Free thyroxine.

Test	Value	Units	Reference
Leucocyte count	4.3	× 10^9^/L	3.3-8.6
Neutrocyte	46	%	43.0-65.0
Lymphocyte	48	%	20.0-50.0
Hemoglobin	14.4	g/dL	11.6-14.8
Thrombocyte count	217	× 10^9^/L	158-348
Total protein	7.6	g/dL	6.6­-8.1
Albumin	5.6	g/dL	4.1-5.1
AST	40	U/L	13­-30
ALT	64	U/L	23-Jul
Uric nitrogen	15.5	mg/dL	20-Aug
Creatinine	0.63	mg/dL	0.46-0.79
Cholesterol	602	mg/dL	142-248
Triglyceride	207	mg/dL	30-117
HDLC	89	mg/dL	48-103
Sodium	139	mmol/L	138-145
Potassium	4.6	mmol/L	3.6-4.8
Chloride	103	mmol/L	101-108
Calcium	10.3	mg/dL	8.8-10.1
Phosphorus	3.1	mg/dL	2.7-4.6
Magnesium	2.1	mg/dL	1.8-2.4
C-reactive protein	0.01	mg/dL	0.0-0.14
TSH	5.683	μIU/mL	0.35-4.95
FT_3_	1.41	pg/mL	1.68-3.67
FT_4_	0.97	ng/dL	0.7-1.48
Ferritin	151.3	ng/mL	5-152
Vitamin B_1_	31.4	ng/mL	21.3-81.9
Retinol-binding protein	4.4	mg/dL	2.5-7.1
Prealbumin	26.2	mg/dL	22-34

Chest X-ray revealed a cardiothoracic ratio of 37% and a teardrop-shaped heart. ECG showed low voltages. Echocardiography showed a small amount of pericardial effusion, but it was asymptomatic (i.e., no cardiac tamponade was noted). MRI of the head showed atrophy of the brain parenchyma and a lack of subcutaneous fat around the orbit and face. The anterior pituitary gland was relatively small for such an age, suggesting malnutrition. We initially diagnosed AN because she met all the DSM-5 diagnostic criteria for AN. Based on the patient’s BMI of <15 kg/m2, she was classified as severely underweight. However, she understood the importance of nutrition well and ate all the food she was given (2300 kcal) on the first day of admission. She did not seem to fear gaining weight and desired to be slim. Thus, we reconsidered our preliminary diagnosis of AN.

**Figure 1:**
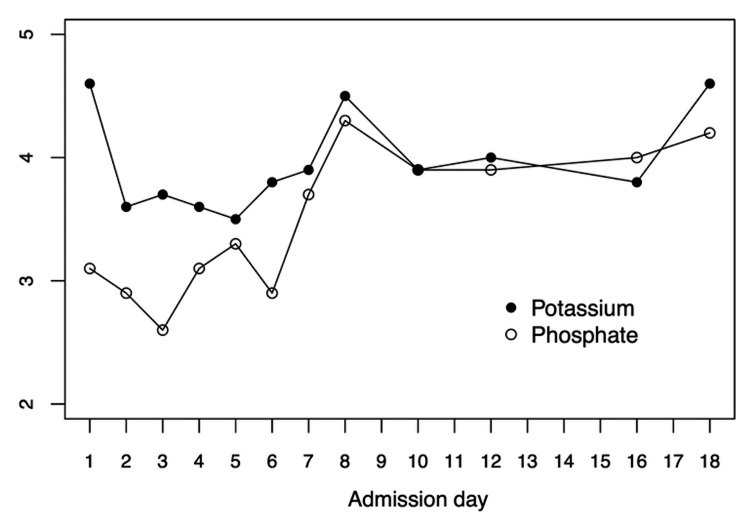
Changes in the value of potassium and of phosphate in the clinical course. The unit of potassium is mmol/L, and that of phosphate is mg/dL.

Avoidant/restrictive food intake disorder (ARFID) was excluded because she exhibited no food-avoidance behaviors. Prior to admission, she exhibited selective eating behaviors, such as voluntarily eating almost exclusively vegetables without carbohydrates or proteins. Thus, we suspected ON, an eating disorder characterized by an obsession with healthy nutrition. Although we were concerned about refeeding syndrome, we continued to provide a diet of 2300 kcal per day to maintain her motivation to eat. Daily blood tests were performed to monitor her potassium and phosphorus levels. On the second day of admission, oral phosphorus was administered because a phosphorus level of 2.6 mg/dL was noted. On the fifth day of admission, oral potassium chloride was administered because the value of potassium had dropped to 3.5 mEq/dL. The oral phosphorus and potassium were tapered from the 8th day and finished on the 16th day (Figure 1).

We performed follow-up echocardiography on the ninth day, but there were no changes in the pericardial effusion. However, we did not perform any additional interventions because it was asymptomatic. The patient seemed to have difficulty communicating at admission, so we performed a Rosenzweig picture-frustration study [3], a psychological test to assess her personality. The group concordance rate was 62%, which was within the normal range for her age group. However, the psychologists who examined her pointed out her poor ability for self-reflection, low tolerance for blame, and difficulty reading the context of words. Because of her gradual weight gain (weight of 28.2 kg) and a BMI of 13.1, and the apparent improvement in symptoms at initial presentation, such as lethargy, mood discomfort, and cold extremities, she was discharged from the hospital on the 18th day of admission. At eight months after discharge, her height and weight had increased to 148 cm and 32.9 kg, respectively, and her BMI was 15.0, but her menstruation had not resumed. The difficulties in communicating that were noted during hospitalization improved after discharge, and we interpreted it as a transient condition brought about by brain atrophy due to undernutrition. After her symptoms subsided, we interviewed her and her mother regarding the reason for her sudden weight loss. There were no traumatic episodes, and she had followed a diet that was mainly vegetable-based and devoid of carbohydrates and protein. She had initially read an article on such a diet in some teen magazines, which led her to search for further information on YouTube and TikTok, and she became obsessed with the diet. Until her hospitalization, she had stubbornly adhered to the diet, which consisted of a small amount of low-carbohydrate bread in the morning, a packed lunch she made by herself consisting almost entirely of vegetables, and only vegetables for dinner. Her calorie intake at that time was estimated to be approximately 500 kcal per day. We administered the ORTO-15 [4], which determines the propensity for ON, and her score of 39 points satisfied the diagnostic criteria for ON.

## Discussion

The DSM-5, currently the most commonly used classification of eating disorders, defines the diagnostic criteria for AN as follows [5]: (A) Restriction of energy intake relative to requirements, leading to significantly low body weight in the context of the age, sex, developmental trajectory, and physical health (less than minimally normal/expected); (B) Intense fear of gaining weight or becoming fat or persistent behavior that interferes with weight gain; (C) Disturbed by one’s body weight or shape, self-worth influenced by body weight or shape, or persistent lack of recognition of seriousness of low body weight.

Although this patient satisfied all the diagnostic criteria, namely restriction of energy intake, persistent behaviors that interfered with weight gain, and a lack of recognition of the seriousness of low body weight, she did not exhibit an intense fear of gaining weight, and her body weight or shape did not influence her self-worth. Furthermore, she did not appear to have a distorted perception of her weight or body shape, a specific characteristic of AN [6]. In the previous DSM-IV classification, eating disorders were classified into AN, BN, and eating disorders not otherwise specified (EDNOS). Although 62.4% of eating disorder cases among patients aged 8-19 years were classified as EDNOS according to the DSM-IV, the DSM-5 was revised to omit EDNOS [7]. In the DSM-5, pica, rumination disorder, and ARFID, which were originally classified under infantile or early childhood feeding and eating disorders, were newly included as eating disorders. Suppose we apply this disease classification to the present case. In that case, it might fall under the category of ARFID, the diagnostic criteria of which are as follows: (A) An eating or feeding disturbance (e.g., apparent lack of interest in eating or food; avoidance based on the sensory characteristics of food; concern about aversive consequences of eating) as manifested by persistent failure to meet appropriate nutritional and/or energy needs associated with one (or more) of the following: 1. Significant weight loss (or failure to achieve expected weight gain or faltering growth in children; 2. Significant nutritional deficiency; 3. Dependence on enteral feeding or oral nutritional supplements; 4. Marked interference with psychosocial functioning; (B) The disturbance is not better explained by lack of available food or by an associated culturally sanctioned practice; (C) The eating disturbance does not occur exclusively during the course of AN or BN, and there is no evidence of a disturbance in the way in which one’s body weight or shape is experienced; (D) The eating disturbance is not attributable to a concurrent medical condition or not better explained by another mental disorder. When the eating disturbance occurs in the context of another mental disorder, the severity of the eating disturbance exceeds that routinely associated with the condition or disorder and warrants additional clinical attention.

**Figure 2:**
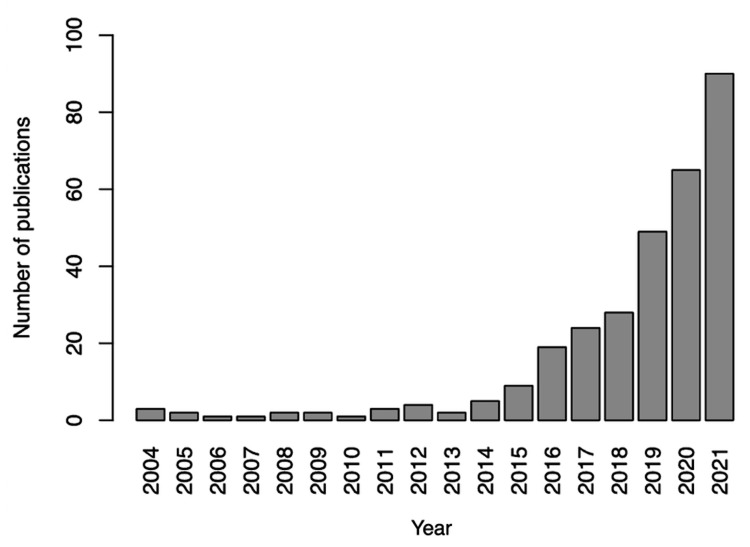
Trends in the number of publications on orthorexia nervosa. We searched PubMed for the term “Orthorexia nervosa,” and counted the annual number of publications.

However, the DSM-5 gives three examples of ARFID: apparent indifference to eating or food, avoidance based on sensory characteristics of food, and anxiety about aversive consequences after eating. These examples suggest that the concept of ARFID is based on an objection to food or eating behaviors. Therefore, we considered that this case was not ARFID because the patient did not refuse food or object to eating behaviors but was unwilling to eat of her own volition. ON is a disease first proposed by Bratman and Knight in 1997 to describe cases of obsession with healthy nutrition management [8]. Although the disease is not included in the DSM-5, it has been increasingly recognized in recent years. A search for “orthorexia nervosa” in PubMed shows a gradual increase in the number of published papers since 2015, with a rapid increase in recent years (Figure 2).

According to a survey in the Netherlands, 78% of psychiatrists, psychologists, nutritionists, and therapists regard ON as an independent disease, and 74% favor its inclusion in the DSM [9]. Although the diagnostic criteria for ON have not been strictly defined, the concept of ON is based on food avoidance due to a preoccupation with healthy foods, as listed [10]: (A) obsessive or pathological preoccupation with healthy nutrition; (B) distress or anxiety caused by failure to follow self-imposed rules about eating; (C) psychosocial disturbances in life in addition to malnutrition and weight loss.

**Figure 3:**
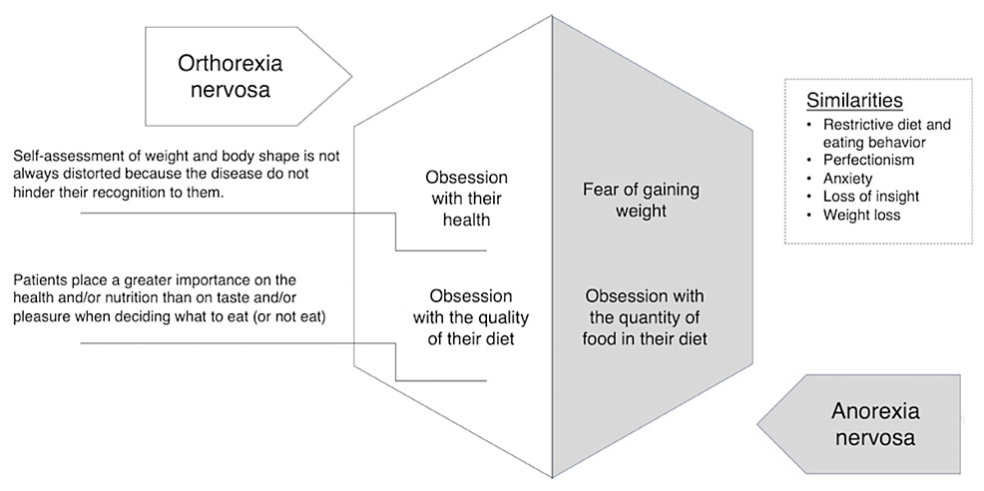
Differences and similarities between anorexia nervosa and orthorexia nervosa.

The ORTO-15 is commonly used as a diagnostic tool for ON [4]. Lower scores suggest the temperament of ON, and this case satisfied the diagnostic criteria for ON with a score of 39 points (<40 points). In this case, the patient showed deep empathy with choosing foods based on calorie content, spending money on healthy foods, and choosing foods based on her health status. Patients with AN tend to hide their eating behavior, while patients with ON tend to flaunt their behavior because of their strong health awareness [11]. In this case, the patient also promoted the benefits of her eating habits to the medical staff. The differences between AN and ON are summarized in Figure 3. Because the treatment approaches may differ [9], it is essential to distinguish between AN and ON.

It has been reported that the incidence of various eating disorders increased during the COVID-19 pandemic [12]. Indeed, ON has increased by 67% in males and 83% in females [13]. Instagram is one of the most popular social media services used by young people, and a significant correlation between ORTO-15 scores and Instagram use has been reported [14]. Thus, the increase in time spent on social media due to the COVID-19 pandemic is considered one of the reasons for the increase in ON in the pandemic era. The patient was exposed to teen magazine articles and social media posts promoting the vegetable-based diet, which led her to research it further on other social media platforms, such as YouTube and TikTok, and she stubbornly adhered to the diet thereafter. Although the ability for children to freely access information online is not inherently negative, there is always a risk of exposure to misinformation. In this case, exposure to misinformation negatively affected her health and growth. Pediatricians should stay up to date on youth diet trends and work to combat misinformation, and explain the nutritional intake necessary for them to grow and lead healthy lives. This goal might also be accomplished through collaboration with schools.

## Conclusions

We reported the case of a 13-year-old girl with ON, whose unhealthy dietary choices were influenced by the information she read in magazines and on social media. Pediatricians should raise awareness of misinformation about children’s health to ensure that they grow and lead healthy lives, especially during the COVID-19 pandemic, which has led to increased use of social media by young people.

## References

[1] van Hoeken D, Hoek HW (2020). Review of the burden of eating disorders: mortality, disability, costs, quality of life, and family burden. Curr Opin Psychiatry.

[2] Swanson SA, Crow SJ, Le Grange D, Swendsen J, Merikangas KR (2011). Prevalence and correlates of eating disorders in adolescents. Results from the national comorbidity survey replication adolescent supplement. Arch Gen Psychiatry.

[3] Harrison A, Genders R, Davies H, Treasure J, Tchanturia K (2011). Experimental measurement of the regulation of anger and aggression in women with anorexia nervosa. Clin Psychol Psychother.

[4] Heiss S, Coffino JA, Hormes JM (2019). What does the ORTO-15 measure? Assessing the construct validity of a common orthorexia nervosa questionnaire in a meat avoiding sample. Appetite.

[5] Call C, Walsh BT, Attia E (2013). From DSM-IV to DSM-5: changes to eating disorder diagnoses. Curr Opin Psychiatry.

[6] Cooper MJ, Fairburn CG (1992). Thoughts about eating, weight and shape in anorexia nervosa and bulimia nervosa. Behav Res Ther.

[7] Peebles R, Hardy KK, Wilson JL, Lock JD (2010). Are diagnostic criteria for eating disorders markers of medical severity?. Pediatrics.

[8] Hanganu-Bresch C (2020). Orthorexia: eating right in the context of healthism. Med Humanit.

[9] Ryman FV, Cesuroglu T, Bood ZM, Syurina EV (2019). Orthorexia nervosa: disorder or not? Opinions of Dutch health professionals. Front Psychol.

[10] Dunn TM, Bratman S (2016). On orthorexia nervosa: a review of the literature and proposed diagnostic criteria. Eat Behav.

[11] Koven NS, Abry AW (2015). The clinical basis of orthorexia nervosa: emerging perspectives. Neuropsychiatr Dis Treat.

[12] J Devoe D, Han A, Anderson A (2022). The impact of the COVID-19 pandemic on eating disorders: a systematic review. Int J Eat Disord.

[13] Kuśnierz C, Rogowska AM, Kwaśnicka A, Ochnik D (2021). The mediating role of orthorexia in the relationship between physical activity and fear of COVID-19 among university students in Poland. J Clin Med.

[14] Turner PG, Lefevre CE (2017). Instagram use is linked to increased symptoms of orthorexia nervosa. Eat Weight Disord.

